# BYSL Promotes Glioblastoma Cell Migration, Invasion, and Mesenchymal Transition Through the GSK-3β/β-Catenin Signaling Pathway

**DOI:** 10.3389/fonc.2020.565225

**Published:** 2020-10-15

**Authors:** Zhuang Sha, Junbo Zhou, Yihao Wu, Tong Zhang, Cheng Li, Qingming Meng, Preethi Priyanka Musunuru, Fangting You, Yue Wu, Rutong Yu, Shangfeng Gao

**Affiliations:** ^1^Institute of Nervous System Diseases, The Affiliated Hospital of Xuzhou Medical University, Xuzhou Medical University, Xuzhou, China; ^2^Department of Neurosurgery, The Affiliated Hospital of Xuzhou Medical University, Xuzhou, China

**Keywords:** bystin, glioma, migration, invasion, GSK-3β, EMT

## Abstract

*BYSL*, which encodes the human bystin protein, is a sensitive marker for astrocyte proliferation during brain damage and inflammation. Previous studies have revealed that BYSL has important roles in embryo implantation and prostate cancer infiltration. However, the role and mechanism of BYSL in glioblastoma (GBM) cell migration and invasion remain unknown. We found that knockdown of BYSL inhibited cell migration and invasion, downregulated the expression of mesenchymal markers (e.g., β-catenin and N-cadherin), and upregulated the expression of epithelial marker E-cadherin in GBM cell lines. Overexpression of BYSL promoted GBM cell migration, invasion, and epithelial-mesenchymal transition (EMT). In addition, the role of BYSL in promoting EMT was further confirmed in a glioma stem cell line derived from a GBM patient. Mechanistically, overexpression of BYSL increased the phosphorylation of GSK-3β and the nuclear distribution of β-catenin. Inhibition of GSK-3β by 1-Azakenpaullone could partially reverse the effects of BYSL downregulation on the transcriptional activity of β-catenin, the expression of EMT markers, and GBM cell migration/invasion. Moreover, immunohistochemical analysis showed strong expression of BYSL in GBM tissues, which was positively correlated with markers of mesenchymal GBM. These results suggest that BYSL promotes GBM cell migration, invasion, and EMT through the GSK-3β/β-catenin signaling pathway.

## Introduction

Glioma is the most common tumor of the central nervous system. Despite comprehensive treatments (surgical resection and chemoradiotherapy) to improve patient prognosis, the overall survival of patients with glioblastoma (GBM) remains poor (1–4), and aggressive growth and unregulated proliferation contribute to poor efficacy of treatment. Therefore, investigation of novel therapeutic targets to combat tumor growth and expansion is critical to improve the treatment of this currently incurable type of cancer.

*BYSL*, which encodes the bystin protein, is a highly conserved gene that has evolved from yeast to humans (5, 6). In humans, BYSL, together with adhesion molecules trophinin and tastin, forms a complex that is highly expressed in trophoblast cells and endometrial cells of the utero-placental interface in early pregnancy and disappears in the second trimester of pregnancy (7). When embryos are transplanted, trophoblast cells actively proliferate and invade the uterine wall, promoting placenta formation and embryo implantation (8). This process is very similar to that of tumor invasion of surrounding tissues. It has been reported that BYSL has an oncogenic role in breast, prostate, liver, and ovarian cancer (9–12). Importantly, BYSL is highly expressed in neural infiltration models of prostate cancer (12).

Epithelial-mesenchymal transition (EMT) is a reversible biological process characterized by loss of polarized organization and acquisition of migratory and invasive capabilities (13, 14). Verhaak et al. classified GBM into four subtypes, proneural, neural, classical, and mesenchymal. The mesenchymal subtype is characterized by strong expression of mesenchymal markers (CHI3L1 and CD44) (15). These markers are reminiscent of an EMT that has been linked to dedifferentiated and transdifferentiated tumors (16).

WNT and β-catenin are highly expressed in GBM tissues and are associated with poor prognosis in patients with GBM (4, 17). The activation of WNT/β-catenin leads to inhibition of the axin complex (axin/APC/CK1/GSK-3β) and thus to the stabilization of β-catenin. The accumulated β-catenin translocates to the nucleus and activates the transcription of target genes, including Twist1/2, MMP7, and Survivin (18). WNT/β-catenin signaling is involved in glioma cell invasion and EMT (19, 20).

In this study, we hypothesized that BYSL might contribute to GBM cell migration, invasion, and EMT *via* GSK-3β/β-catenin signaling. We first investigated the role of BYSL in cell migration, invasion, and EMT in GBM cell lines using small interfering RNA (siRNA) and a lentivirus overexpressing BYSL. Then, we confirmed the promotion of EMT by BYSL in glioma stem cells (GSCs). Finally, we used 1-Azakenpaullone (a GSK-3β inhibitor) to demonstrate the necessity of GSK-3β activity in the regulation by BYSL of GBM cell migration, invasion, and EMT. In addition, clinical samples were used to detect the expression of BYSL in non-tumor brain tissues and GBM tissues, and to explore the correlation between BYSL and mesenchymal makers (e.g., CHI3L1 and CD44).

## Materials and Methods

### Patients and Samples

All the GBM tissue specimens (obtained during surgical resection) and non-tumor brain tissue specimens (obtained from patients undergoing surgery for internal decompression after cerebral trauma) were collected from the Affiliated Hospital of Xuzhou Medical University. All the patients were naïve to immunotherapy, radiation, and chemotherapy. The specimens were fixed in 10% buffered formalin and embedded in paraffin for sectioning. Clinicopathological information for all participants is presented in Supplementary Table 1. All the GBM specimens were from patients with a confirmed pathological diagnosis, classified according to the criteria of the World Health Organization.

### Cell Lines and Cell Culture

HEK 293T cells and human GBM cell lines U251 and U87 were purchased from the Shanghai Cell Bank, Type Culture Collection Committee, Chinese Academy of Sciences. The identities of the U251 and U87 cell lines were confirmed by DNA profiling test (STR). Cells were grown in Dulbecco's modified Eagle's medium (HEK 293T and U251) or minimal essential medium (U87) supplemented with 10% fetal bovine serum (Thermo Fisher Scientific, Waltham, MA). All cell lines were cultured in a cell incubator with a 5% CO_2_ atmosphere under saturated humidity at 37 °C.

### Reagents, Antibodies, and Plasmids

1-Azakenpaullone (1-Az, Selleck, Shanghai, S7193), Lipofectamine 2000 (Invitrogen, Carlsbad, CA), and PolyJet (SignaGen, Gaithersburg, MD) were purchased from the corresponding companies. The primary antibodies used for western blot were as follows: BYSL (1:500, Sigma, St. Louis, MO, HPA031217), β-catenin (1:2000, Cell Signaling Technology, Denver, CO, 8480s), N-cadherin (1:1000, Abcam, Cambridge, UK, ab98952), E-cadherin (1:1000, Proteintech, Rosemont, IL, 20874-1-AP), Slug (1:1000, Abcam, ab180714), Vimentin (1:1000, Santa Cruz Bio, Santa Cruz, CA, sc-373717), GSK-3β (1:2000, Cell Signaling Technology, 9832S), p-GSK-3β (1:2000, Cell Signaling Technology, 9323T), Flag (1:1000, Sigma, F1804), β-actin (1:1000, Santa Cruz Bio, sc-47778), GAPDH (1:20000, Proteintech, 60004-1-Ig), Histone H3 (1:1000, Cell Signaling Technology, 4499S). The Flag-tagged BYSL-overexpressing plasmid was purchased from Viogene Biosciences (Jinan, Shandong, China). TOP-Flash, FOP-Flash, and pGMLR-TK plasmids were obtained from GenScript (Hong Kong, China).

### Transfection

For siRNA transfection, a previously validated BYSL siRNA (10) was synthesized by Biomics Biotech (Nantong, China). Cells were seeded in six-well plates at 50–70% confluence, and BYSL siRNA (siBYSL, 100 nM) or negative control (siNC, 100 nM) was transfected using Lipofectamine 2000 according to the protocol provided by the manufacturer.

For plasmid transfection, when the cells had grown to 70–90% confluence on a 6-cm plate, the plasmid (1 μg) was transfected using PolyJet (3 μL) according to the manufacturer's instructions.

### Lentivirus Construction, Production, and Infection

Human *BYSL* (accession number: NM_004053) was inserted into the pCDH-GFP-puro vector plasmid at the Nhe I and Bgl II sites. The lentiviruses were produced in HEK293T cells and used to infect GBM cells according to our previously reported protocol (21). Forty-eight hours (h) after infection, the infected cells were cultured in medium containing 2.5 μg/mL puromycin (Sigma) for selection. The surviving cells were used in the subsequent experiments.

### Wound Healing Assay

Cells were seeded in a six-well plate and incubated at 37 °C until they reached 80–90% confluence. A wounding line was scratched with a 200 μL pipette tip, and the dead cells were washed with phosphate-buffered saline (PBS). Then, serum-free DMEM was added to each well. The migrating cells were monitored using an IX-71 inverted microscope (Olympus, Tokyo, Japan). Images were taken in three randomly selected fields at 0, 24, and 48 h. The number of migrating cells was counted based on the captured images using ImageJ software (National Institutes of Health, Bethesda, MD).

### Transwell Assay

To assess cell migration and invasion, a transwell assay was performed in a 24-well chamber system with a polycarbonate membrane (Corning, Corning, NY) as described in the literature (22, 23). Briefly, 200 μL of serum-free medium was added to the upper chamber containing 1 × 10^4^ cells. The lower chamber was filled with 500 μL of medium containing 10% fetal bovine serum and then incubated at 37 °C for 24 or 48 h. To assess invasion ability, Matrigel (BD, Franklin Lakes, NJ) was pre-coated onto the polycarbonate membrane; the rest of the procedure remained the same. The migrating and invading cells were counted on the captured images as described previously (21).

### RNA Extraction and Quantitative Real-Time PCR (qRT-PCR)

Total RNA was extracted from cultured cells using TRIzol (Invitrogen) according to the instructions provided by the manufacturer. We employed a Prime Script RT Reagent Kit (TAKARA, Dalian, China) to perform the reverse transcription. The target gene was amplified in a final volume of 20 μL with a SYBR Green PCR Master mix (TAKARA). The qRT-PCR reaction was run in an Applied Biosystems 7500 Real-Time PCR System (Waltham, MA), and data were collected automatically. Forward and reverse primers for all genes are given in Supplementary Table 2. The expression of target genes was normalized to that of β-actin, and relative absolute amounts of target genes were calculated according to our previous method for statistical analysis (24).

### Protein Extraction and Western Blot

Cells were washed in PBS and lysed in ice-cold lysis buffer to obtain whole-cell protein. Cytoplasmic and nuclear proteins were extracted using a commercial kit (Beyotime Biotech Inc, Nantong, China) according to the manufacturer's instructions. Equal amounts of total protein were loaded for western blot analysis according to a protocol similar to that described in our recently published paper (21). The source and dilution ratio of the primary antibodies were as described in the section “Reagents, antibodies, and plasmids.” Band densities were quantified using the ImageJ software. β-actin or GAPDH was used as a loading control for cytoplasmic protein, and Histone H3 was used as a loading control for nuclear protein.

### GSCs Culture and Neurosphere Formation Assay

Glioma tissue samples were obtained from an IDH1-wildtype GBM patient (male, 53 years old) during surgery. His GSCs (named GSC-F) were established and cultured as previously described (25). In brief, GBM tissues were washed, minced, and enzymatically dissociated, after which the tumor cells were suspended in stem cell medium (SCM) (DMEM/F12 medium with 2% B27 (Thermo Fisher Scientific), 1% N2 (Thermo Fisher Scientific), 20 ng/mL EGF and bFGF (Peprotech, Rocky Hill, NJ), HEPES (final concentration of 5 mM), and 1% penicillin/streptomycin solution. The neurosphere formation assay was performed as follows. GSC-F cells were dissociated into single cells at a concentration of 10,000–50,000 cells/mL, and 200 μL of the cell suspension was added to each well of a 96-well plate. The SCM was replaced every 3–4 days. After 6–8 days, neurospheres (diameter ≥ 50 μm) in each well were counted.

### TOP/FOP-Flash Reporter Assay

We used a TOP/FOP-Flash reporter assay to detect the transcriptional activity of β-catenin as described previously (19). Briefly, cells were seeded in a 96-well plate and transfected with siBYSL or siNC in the presence of reporter plasmids containing TOP-Flash or mutated FOP-Flash TCF/LEF DNA-binding sites and pGMLR-TK plasmid. The groups were as follows: siNC + TOP-Flash + pGMLR-TK + DMSO group, siNC + FOP-Flash + pGMLR-TK + DMSO group, siBYSL + TOP-Flash + pGMLR-TK + DMSO group, siBYSL + FOP-Flash + pGMLR-TK + DMSO group, siNC + TOP-Flash + pGMLR-TK + 1-Az group, siNC + FOP-Flash + pGMLR-TK + 1-Az group, siBYSL + TOP-Flash + pGMLR-TK + 1-Az group, siBYSL + FOP-Flash + pGMLR-TK + 1-Az group. A dual luciferase reporter assay system (Promega, Madison, WI) was used to measure luciferase activity 24 h after transfection. The luciferase activity of each sample was normalized to the respective Renilla luciferase activity.

### 5-Ethynyl-20-Deoxyuridine (EdU) Incorporation Assay

The EdU assay was performed using a commercial kit (RiboBio, Guangzhou, China), as described in the literature (26). The percentage of EdU-positive cells was calculated by dividing the number of EdU-positive cells by the number of Hoechst-stained cells.

### Immunohistochemistry and Cell Counting

The immunoreactivity (IR) of BYSL, CD44, and CHI3L1 was detected by immunohistochemistry and quantified by cell counting, as described in our previous publications (21, 27). Briefly, antigen retrieval was applied to sections in citrate buffer (pH 6.0) with microwaves. Primary antibodies against BYSL (1:50, Sigma), CD44 (1:100, OriGene, Rockville, MD), and CHI3L1 (1:50, Proteintech) were added. All sections were then processed using an ABC Elite kit (Vector Laboratories, Burlingame, CA) according to the manufacturer's protocol. Finally, the sections were counterstained with hematoxylin (KeyGEN BioTECH, Jiangsu, China). All images were captured using a DM2500 microscope (Leica, Wetzlar, Germany), and cell counting was performed by an investigator without knowledge of the identity of any of the subjects.

### Statistical Analysis

*In vitro* experiments were repeated at least three times, and data are expressed as mean ± S.D. Comparisons between two groups were performed by Student's *t*-test. Differences among multiple groups were determined by one-way analysis of variance followed by Dunnett's or Tukey *post hoc* test. Correlations were analyzed by Spearman correlation test. Statistical analyses were performed using SPSS version 19.0 (SPSS Inc., Chicago, IL). Tests were two-tailed, and values of *P* < 0.05 were considered to be statistically significant.

## Results

### Downregulation of BYSL Inhibits GBM Cell Migration and Invasion

**Figure 1:**
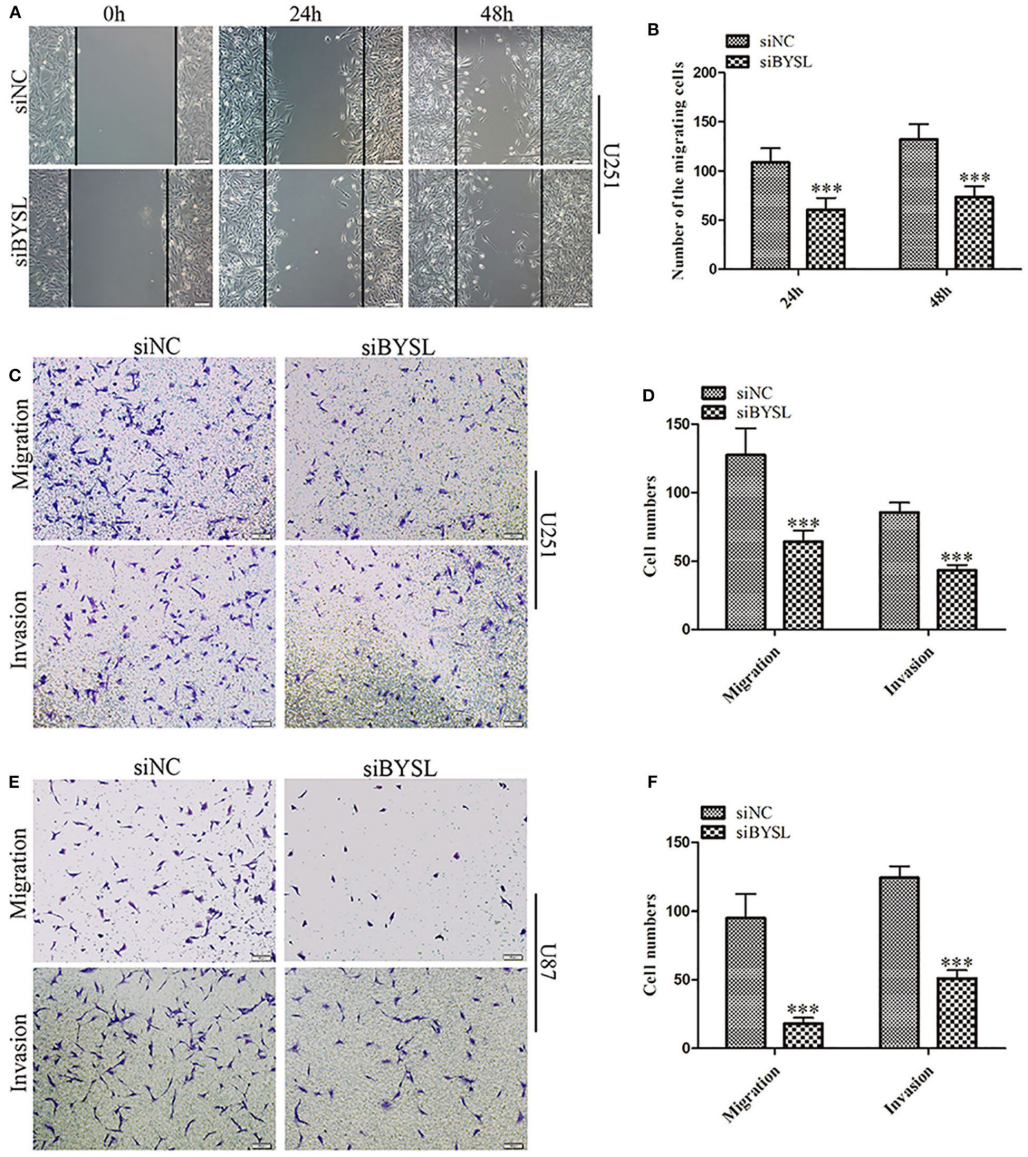
Downregulation of BYSL inhibits GBM cell migration and invasion. **(A,B)** Wound healing assay to assess the effects of BYSL downregulation on cell migration at 24 and 48 h in U251 cells. Representative images are shown in **(A)**, and quantitative analyses of the number of migrating cells are shown in **(B)**. **(C–F)** Transwell assay to evaluate the effects of BYSL downregulation on cell migration and invasion in U251 and U87 cells. Representative images are shown in **(C,E)**, and quantitative analyses of the number of cells migrating to the chamber (migration) or crossing the Matrigel (invasion) are shown in **(D,F)**. Scale bars: 100 μm. ***P < 0.001.

We used a previously validated siRNA for targeting BYSL (10). Western blot and qRT-PCR analyses showed that BYSL was successfully downregulated by the siRNA in both U251 and U87 cells (Supplementary Figure 1). Wound healing and transwell assays were used to assess the effects of downregulation of BYSL on GBM cell migration and invasion. The results of the wound healing assay showed that knockdown of BYSL led to a significant ~40% decrease in the number of migrating cells at 24 and 48 h (all *P* < 0.001) in U251 cells (Figures 1A,B). The transwell assay showed that the numbers of cells migrating to the chamber and crossing the Matrigel were significantly decreased in U251 (percent-change ~50%, all *P* < 0.001) and U87 (percent-change > 60%, all *P* < 0.001) cells after BYSL was downregulated (Figures 1C–F). These results suggest that downregulation of BYSL inhibits GBM cell migration and invasion.

### Downregulation of BYSL Inhibits the EMT in GBM Cells

**Figure 2:**
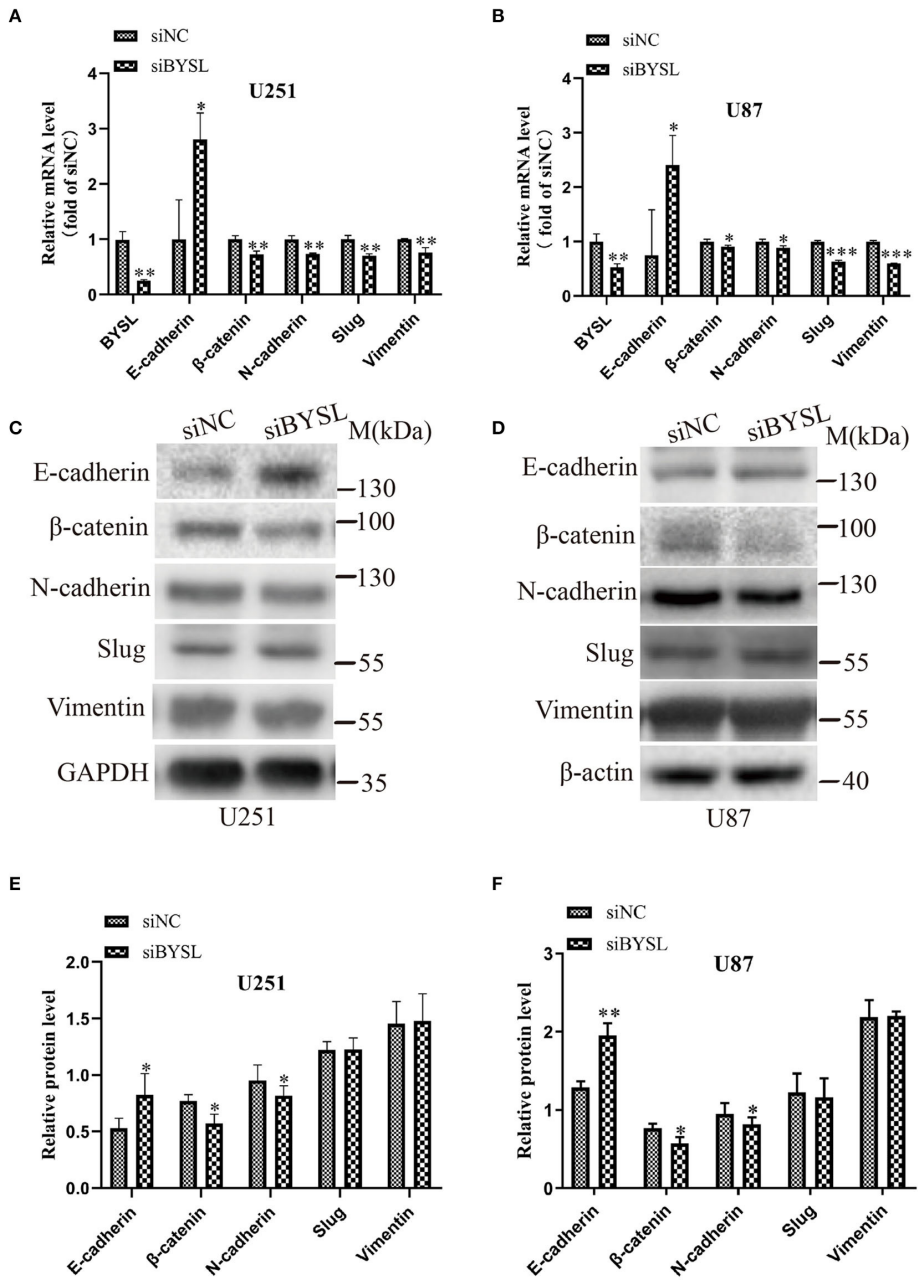
Downregulation of BYSL inhibits the EMT in GBM cells. **(A,B)** QRT-PCR assay to measure changes in the mRNA levels of EMT markers (E-cadherin, β-catenin, N-cadherin, Slug, and Vimentin) in U251 and U87 cells following BYSL downregulation. **(C–F)** Western blot analyses to determine changes in the protein levels of EMT markers in U251 and U87 cells after silencing of BYSL. Representative blot images are shown in **(C,D)**. Quantification graphs are shown in **(E,F)**. M, molecular marker. *P < 0.05, **P < 0.01, ***P < 0.001.

As EMT is closely involved in the aggressive growth of GBM (19, 20), we next detected the expression of mesenchymal and epithelial markers in GBM cells. When BYSL was effectively downregulated by the siRNA, the mRNA levels of mesenchymal markers were significantly reduced in U251 cells (β-catenin: *P* = 0.006, N-cadherin: *P* = 0.003, Slug: *P* = 0.003, Vimentin: *P* = 0.011) and U87 cells (β-catenin: *P* = 0.038, N-cadherin: *P* = 0.029, Slug: *P* < 0.001, Vimentin: *P* < 0.001), whereas E-cadherin, a epithelial marker, was significantly upregulated in U251 cells (*P* = 0.022) and U87 cells (*P* = 0.049; Figures 2A,B). Furthermore, we found that knockdown of BYSL caused a significant 20% decrease in the protein levels of β-catenin (*P* = 0.028) and N-cadherin (*P* = 0.039) in U251 cells, a significant 12% decrease in the β-catenin (*P* = 0.017) and N-cadherin (*P* = 0.024) protein levels in U87 cells, and a significant 50% increase in the E-cadherin protein levels (U251: *P* = 0.046, U87: *P* = 0.003), with no significant effects on other mesenchymal markers (Figures 2C–F). These data suggest that downregulation of BYSL suppresses the EMT in GBM cells.

### Overexpression of BYSL Promotes GBM Cell Migration and Invasion

**Figure 3:**
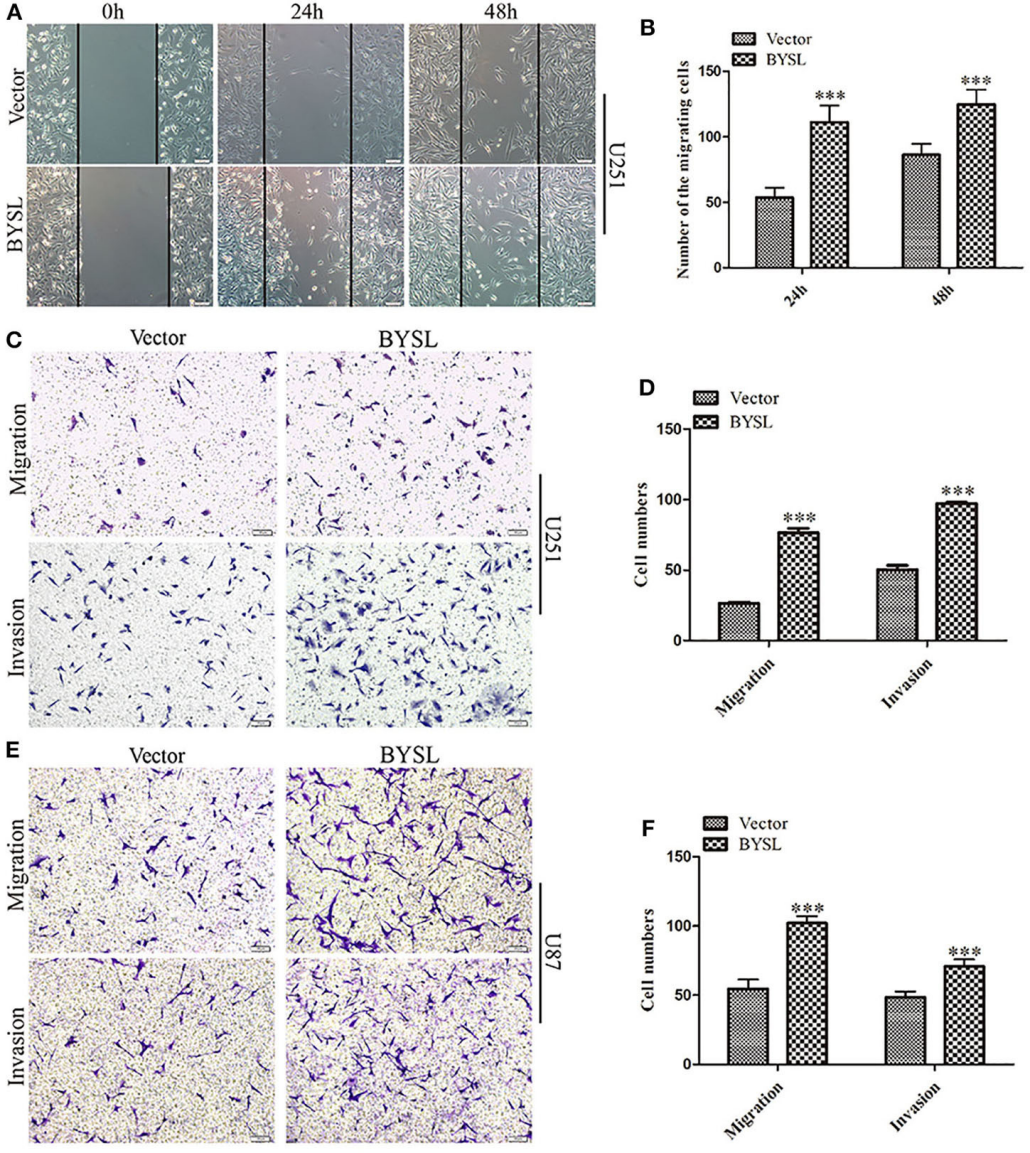
Overexpression of BYSL promotes GBM cell migration and invasion. **(A,B)** Wound healing assay to assess the effects of BYSL overexpression on cell migration at 24 and 48 h in U251 cells. Representative images are shown in **(A)**, and quantitative analyses of the number of migrating cells are shown in **(B)**. **(C–F)** Transwell assay to evaluate the effects of BYSL overexpression on cell migration and invasion in U251 and U87 cells. Representative images are shown in **(C,E)**, and quantitative analyses of the number of cells migrating to the chamber (migration) or crossing the Matrigel (invasion) are shown in **(D,F)**. Scale bars: 100 μm. ***P < 0.001.

We established stable GBM cell lines with overexpression of BYSL by lentivirus-mediated infection in U251 and U87 cells. The number of cells with GFP fluorescence accounted for ~90% of total cells, as observed by fluorescence microscopy. Western blot analysis showed that exogenous BYSL was abundantly overexpressed in U251 and U87 cells (Supplementary Figure 2). Then, wound healing and transwell assays were used to evaluate the influence of BYSL overexpression on the migration and invasion of GBM cells. The wound healing assay showed that the numbers of migrating cells of U251 cells in the BYSL-overexpressing group were increased at 24 h (percent-change ~100%) and 48 h (percent-change ~40%) compared with the vector group (all *P* < 0.001, Figures 3A,B). The transwell assay showed that overexpression of BYSL led to a significant increase in the number of cells migrating to the chamber (percent-change > 85%), and a significant increase in the number of cells crossing the Matrigel (percent-change > 45%) in U251 and U87 cells (all *P* < 0.001, Figures 3C–F). These results indicate that overexpression of BYSL enhances the migration and invasion abilities of GBM cells.

### Overexpression of BYSL Promotes the EMT in GBM Cells

**Figure 4:**
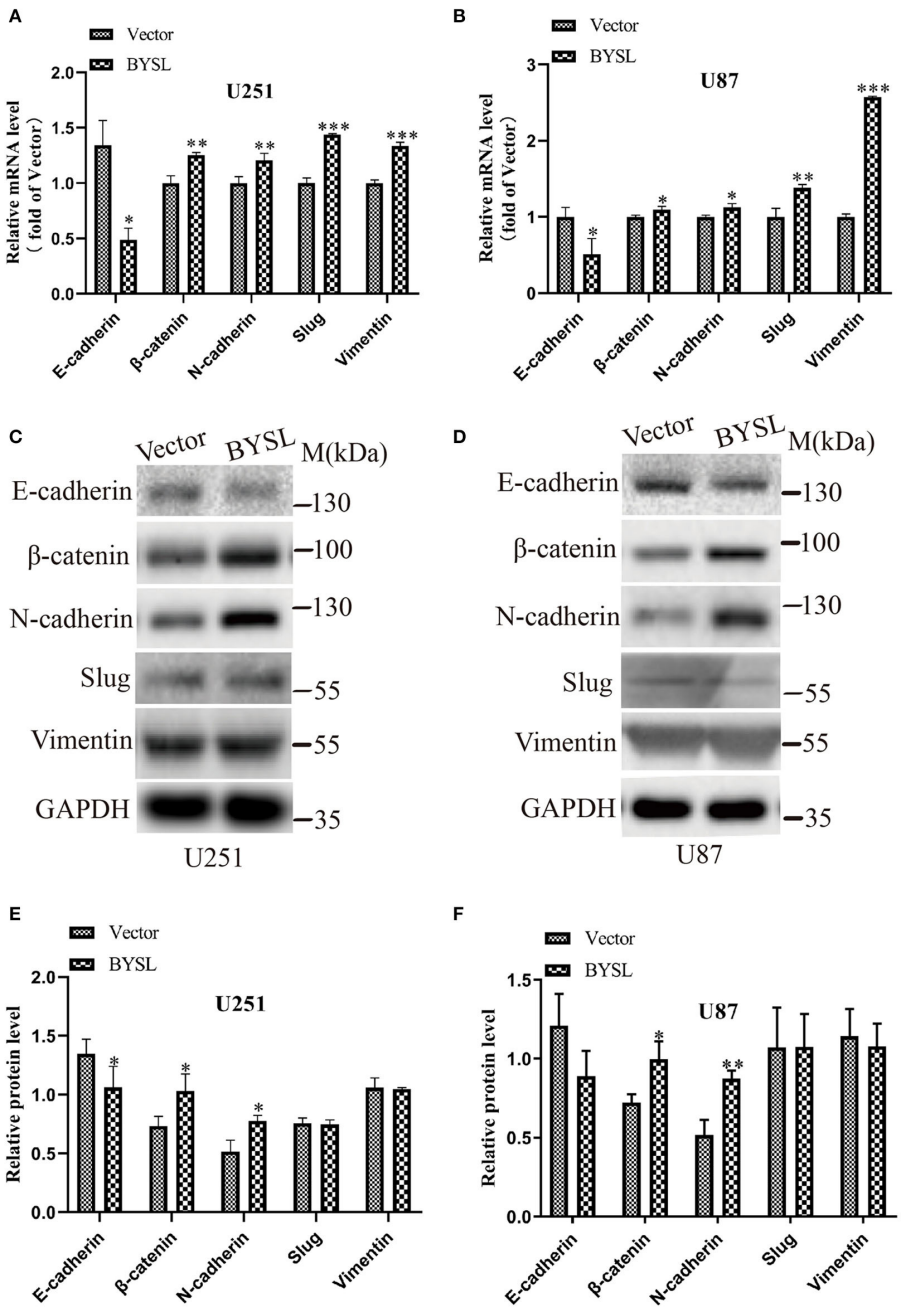
Overexpression of BYSL promotes the EMT in GBM cells. **(A,B)** QRT-PCR assay to measure the mRNA levels of EMT markers (E-cadherin, β-catenin, N-cadherin, Slug, and Vimentin) in U251 and U87 cells following BYSL overexpression. **(C–F)** Western blot analyses to determine the protein expression of EMT markers in BYSL-overexpressing U251 and U87 cells. Representative blot images are shown in **(C,D)**. Quantification graphs are shown in **(E,F)**. M, molecular marker. *P < 0.05, **P < 0.01, ***P < 0.001.

We next used qRT-PCR and western blot analyses to measure the effects of BYSL overexpression on the expression of the EMT markers in GBM cells. The qRT-PCR assay showed that mRNA levels of mesenchymal markers were significantly increased in U251 cells (β-catenin: *P* = 0.004, N-cadherin: *P* = 0.002, Slug: *P* < 0.001, Vimentin: *P* < 0.001) and U87 cells (β-catenin: *P* = 0.033, N-cadherin: *P* = 0.019, Slug: *P* = 0.006, Vimentin: *P* < 0.001), whereas E-cadherin mRNA expression was significantly downregulated (U251: *P* = 0.040; U87: *P* = 0.024) in the BYSL-overexpressing group (Figures 4A,B). Furthermore, overexpression of BYSL significantly increased the protein levels of β-catenin (percent-change ~30%; U251: *P* = 0.045, U87: *P* = 0.019) and N-cadherin (percent-change ~40%; U251: *P* = 0.043, U87: *P* = 0.003), decreased E-cadherin protein levels (percent-change ~20%; U251: *P* = 0.030, U87: *P* = 0.096), and showed no significant effects on other mesenchymal markers (Figures 4C–F). These data suggest that the overexpression of BYSL triggers the expression of EMT activators in GBM cells.

### Downregulation (Overexpression) of BYSL Inhibits (Promotes) Neurosphere Formation and the EMT in GSCs

**Figure 5:**
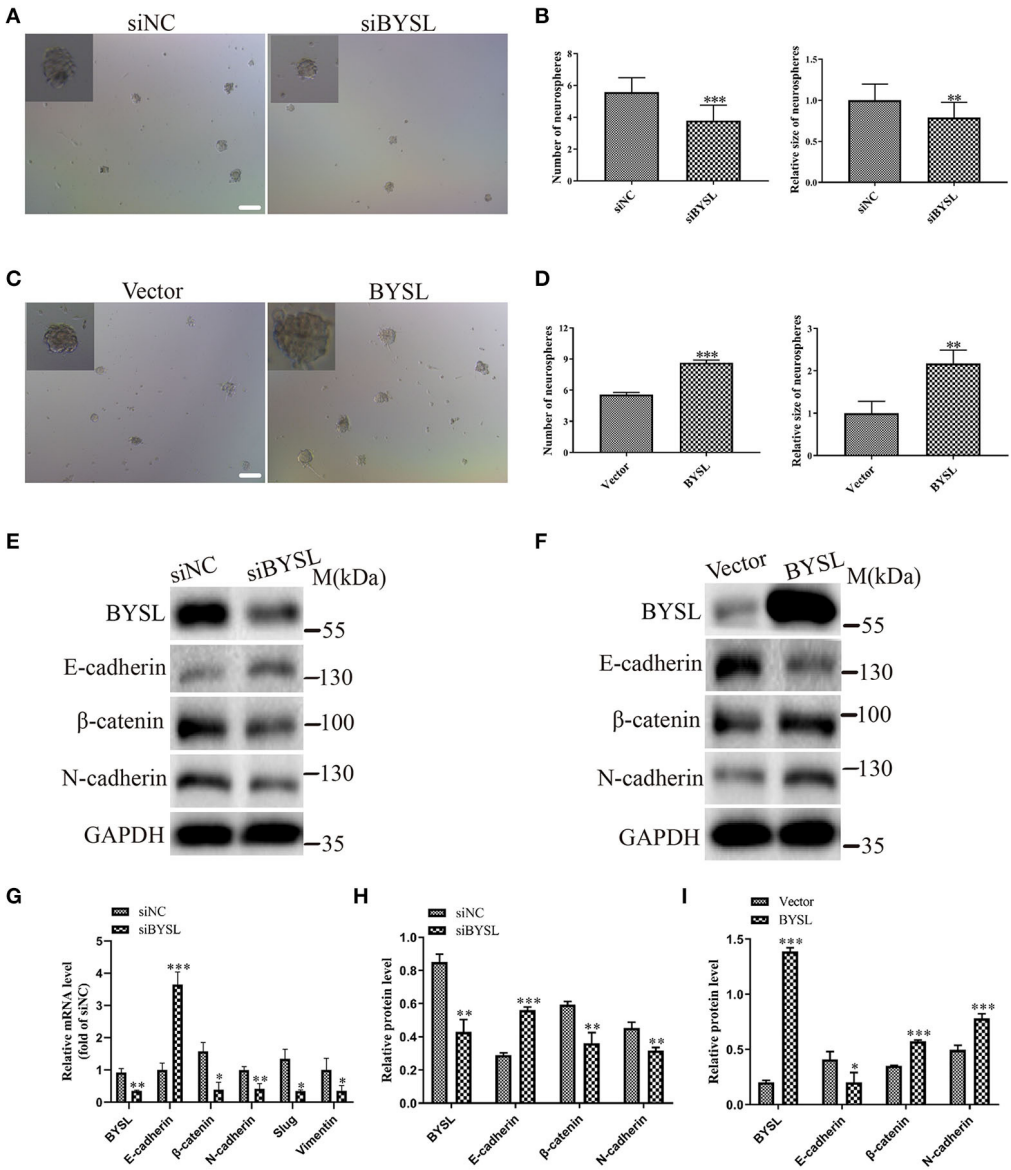
Downregulation (overexpression) of BYSL inhibits (promotes) tumorsphere formation and the EMT process in GSCs. **(A–D)** Neurosphere formation assay in GSC-F cells to evaluate the role of BYSL in neurosphere formation. Representative images are shown in **(A,C)**, and quantitative analyses of the number and size of spheroids are shown in **(B,D)**. Scale bars: 100 μm. **(E–I)** Western blot and qRT-PCR assays to determine the expression changes of EMT markers in the BYSL-silencing/overexpressing GSC-F cells. Representative blot images are shown in **(E,F)**. Quantification graphs are shown in **(G–I)**. M, molecular marker. *P < 0.05, **P < 0.01, ***P < 0.001.

To confirm the role of BYSL in promoting EMT, we performed a neurosphere formation assay and measured the expression of EMT markers in a patient-derived GSC cell line (GSC-F). Immunofluorescence staining showed positive expression of Nestin and CD44 in the GSC-F cells (Supplementary Figure 3). Downregulation of BYSL significantly decreased the number (percent-change ~30%, *P* < 0.001) and size (percent-change ~23%, *P* = 0.002) of neurospheres in the GSCs, whereas overexpression of BYSL showed the opposite effects (Figures 5A–D). Furthermore, the qRT-PCR assay showed that knockdown of BYSL caused significant decreases in mRNA levels of β-catenin (*P* = 0.043), N-cadherin (*P* = 0.007), Slug (*P* = 0.041), and Vimentin (*P* = 0.049), and a significant increase in the E-cadherin mRNA level (*P* < 0.001) in GSC-F cells (Figure 5G). Consistently, there were also significant changes in the protein levels of β-catenin and N-cadherin (percent-change > 30%, all *P* = 0.004), and E-cadherin (percent-change ~50%, *P* < 0.001; Figures 5E,H). On the contrary, overexpression of BYSL caused a significant ~50% increase in the protein levels of β-catenin and N-cadherin (all *P* < 0.001) and a significant ~50% decrease in the E-cadherin protein levels (*P* = 0.032) in GSC-F cells (Figures 5F, I). These results further confirm the role of BYSL in promoting EMT of GBM cells.

### Overexpression of BYSL increases the activity of GSK-3β/β-catenin signaling pathway

**Figure 6:**
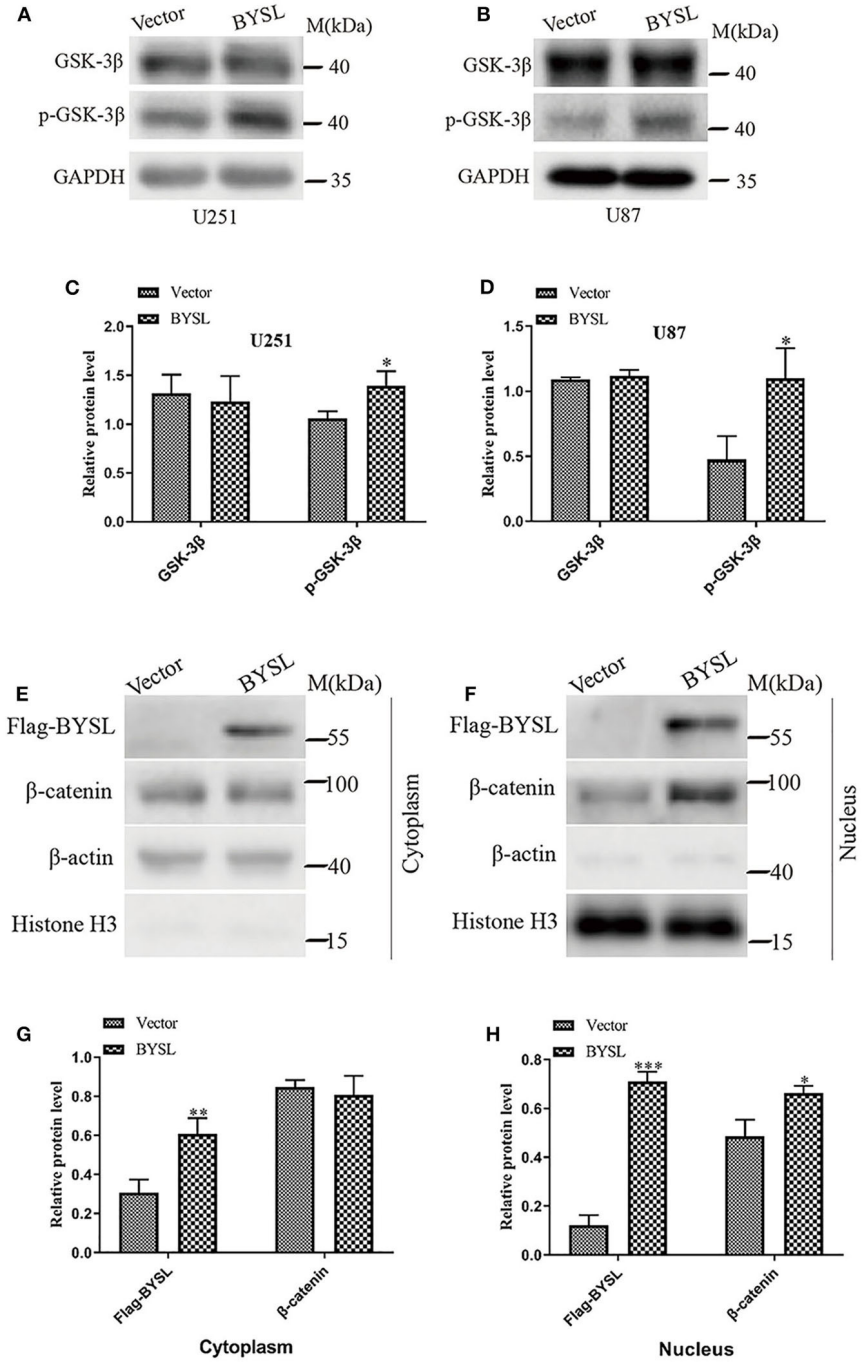
Overexpression of BYSL enhances the activity of GSK-3β/β-catenin signaling pathway in GBM cells. **(A–D)** Western blot analyses to measure the levels of p-GSK-3β and GSK-3β in U251 and U87 cells. Representative blot images are shown in **(A,B)**. Quantification graphs are shown in **(C,D)**. **(E–H)** Distribution of β-catenin in cytoplasm and nucleus as detected by western blot analysis. Representative blot images are shown in **(E,F)**. Quantification graphs are shown in **(G,H)**. M, molecular marker. *P < 0.05, **P < 0.01, ***P < 0.001.

As GSK-3β is an important component of the axin degradation complex, which determines whether β-catenin is transported into the nucleus or undergoes proteasome-dependent degradation (28–31), we next examined the levels of phosphorylated GSK-3β (p-GSK-3β) and total GSK-3β in GBM cell lines. Overexpression of BYSL significantly elevated p-GSK-3β levels in U251 cells (percent-change ~30%, *P* = 0.024) and U87 cells (percent-change ~130%, *P* = 0.025), without affecting the total GSK-3β levels (Figures 6A–D). Moreover, upregulation of BYSL promoted the nuclear distribution of β-catenin in U87 cells (*P* = 0.015, Figures 6E–H). These results imply that GSK-3β/β-catenin signaling is located downstream of BYSL in GBM cells.

### Inhibiting GSK-3β Could Partially Reverse the Diminished β-Catenin Activity Caused by BYSL Downregulation

**Figure 7:**
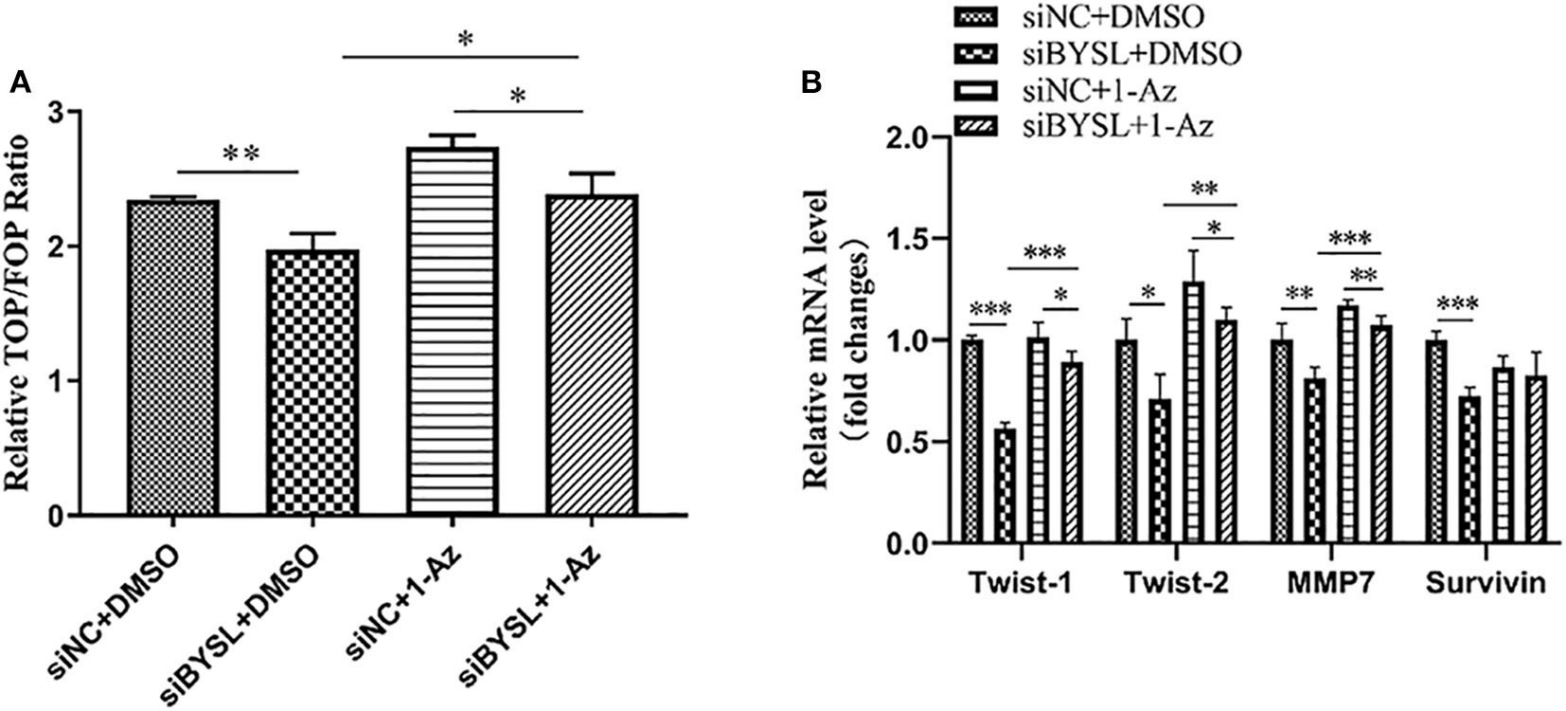
Inhibiting GSK-3β could partially reverse the diminished β-catenin activity caused by BYSL downregulation. **(A)** TOP/FOP-Flash reporter assay to assess the reversal by 1-Az of the reduced transcriptional activity of β-catenin caused by BYSL downregulation in HEK293T cells. **(B)** qRT-PCR assay to evaluate the reversal effects of 1-Az on the decreased mRNA levels of the β-catenin target genes after knockdown of BYSL in U87 cells. *P < 0.05, **P < 0.01, ***P < 0.001.

Consistent with the nuclear translocation of β-catenin following BYSL overexpression, the TOP/FOP-Flash reporter assay showed that the transcriptional activity of β-catenin was significantly repressed in HEK293T cells following knockdown of BYSL (percent-change ~12%, *P* = 0.005, Figure 7A). More importantly, treatment with 1 μM 1-Az (an inhibitor of GSK-3β) reversed the decrease in β-catenin activity caused by downregulation of BYSL (percent-change ~20%, *P* = 0.020, Figure 7A). Furthermore, the qRT-PCR assay showed that the transcription of the β-catenin target genes was significantly reduced following BYSL downregulation (Twist-1: *P* < 0.001, Twist-2: *P* = 0.011, MMP7: *P* = 0.009, Survivin: *P* < 0.001); this reduction was partially reversed by 1-Az administration (Twist-1: *P* < 0.001, Twist-2: *P* = 0.001, MMP7: *P* < 0.001, Survivin: *P* = 0.146) in U87 cells (Figure 7B). These results indicate that GSK-3β activity is required for BYSL-mediated β-catenin activation in GBM cells.

### Inhibiting GSK-3β Could Partially Reverse the Effects of BYSL Downregulation on GBM Cell Migration, Invasion, and EMT

**Figure 8:**
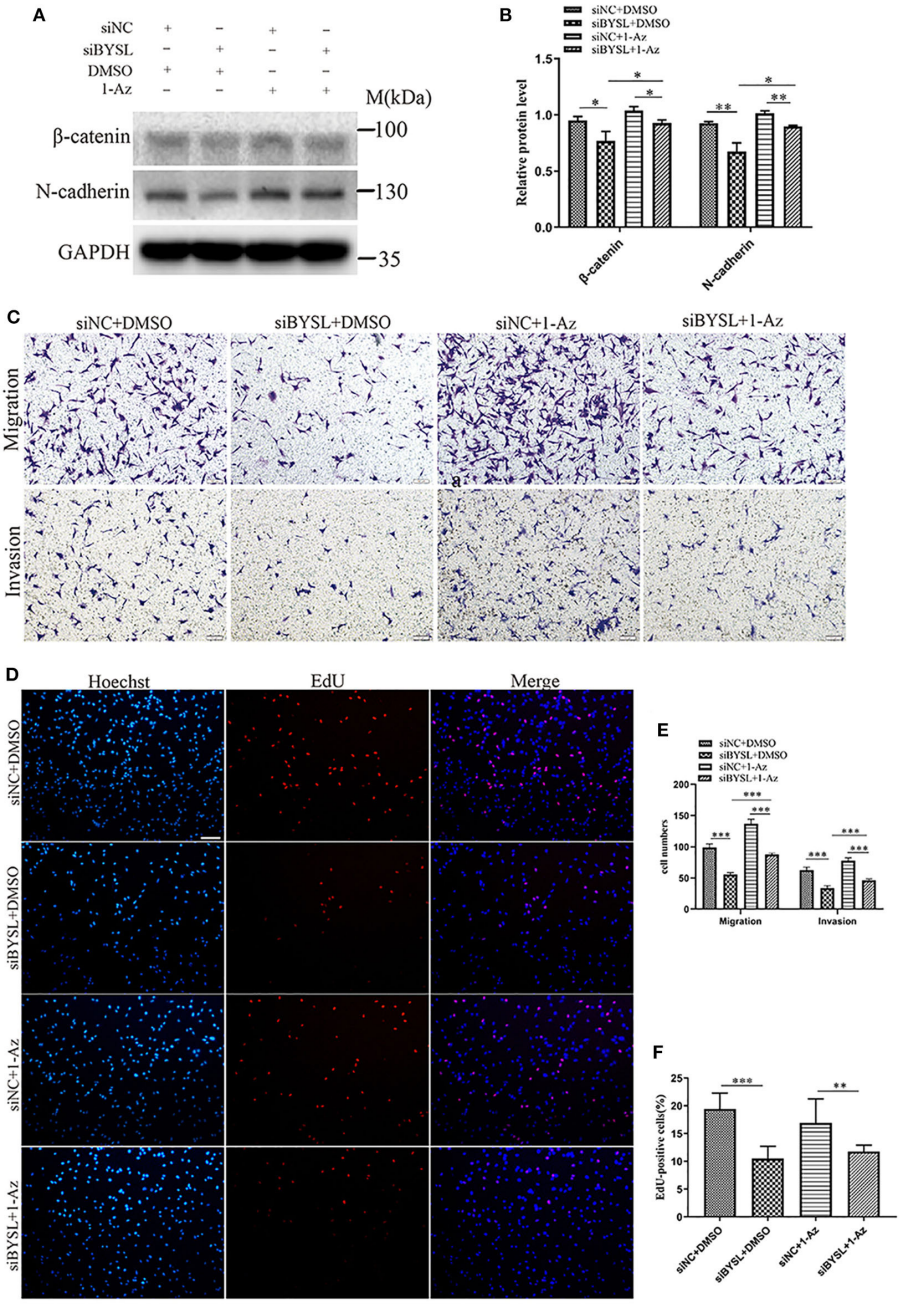
Inhibiting GSK-3β could partially reverse the effects of BYSL downregulation on GBM cell migration, invasion, and EMT. **(A,B)** Western blot analyses to evaluate the reversal by 1-Az of the decrease in β-catenin and N-cadherin levels caused by BYSL downregulation in U87 cells. Representative blot images are shown in **(A)**. Quantification graph is shown in **(B)**. **(C,E)** Transwell assays to assess the reversal by 1-Az of the decreased cell migration and invasion caused by silencing of BYSL in U87 cells. Representative images are shown in **(C)**, and quantitative analyses of the number of cells migrating to the chamber (migration) or crossing the Matrigel (invasion) are shown **(E)**. **(D,F)** EdU assays to evaluate the effects of BYSL downregulation on cell proliferation and the reversal effects mediated by 1-Az treatments in U87 cells. Representative images are shown in **(D)**, and quantitative analyses of the percentages of EdU-positive cells are shown in **(F)**. Scale bars: 100 μm. M, molecular marker. *P < 0.05, **P < 0.01, ***P < 0.001.

Western blot analysis showed that downregulation of BYSL resulted in a significant ~20% decrease in the protein levels of mesenchymal markers (β-catenin: *P* = 0.026, N-cadherin: *P* = 0.005) in U87 cells (Figures 8A,B), consistent with the results shown in Figures 2D–F. Inhibition of GSK-3β by 1-Az partially reversed ~20% decrease in protein levels of mesenchymal markers caused by downregulation of BYSL (β-catenin: *P* = 0.037, N-cadherin: *P* = 0.008). We next used transwell assays to assess the reversal effects of 1-Az on the decrease in cell migration and invasion caused by BYSL downregulation in GBM cells. Consistent with our findings shown in Figures 1E, F, downregulation of BYSL resulted in a significant ~45% decrease in the number of migrating- and invading-U87 cells (all *P* < 0.001, Figures 8C,E). Treatment with 1-Az partially reversed the decrease in migration and invasion ability caused by BYSL downregulation (all *P* < 0.001, Figures 8C,E). In addition, the EdU assay revealed a significant inhibitory effect of BYSL downregulation on the percentage of EdU-positive cells in U87 cells (percent-change ~46%, *P* < 0.001, Figures 8D,F); however, this effect could not be reversed by 1-Az (Figures 8D,F). These data suggest that BYSL promotes GBM cell migration, invasion, and EMT *via* the GSK-3β/β-catenin signaling pathway.

### Strong Expression of BYSL Is Associated With the Mesenchymal GBM Subtype

**Figure 9:**
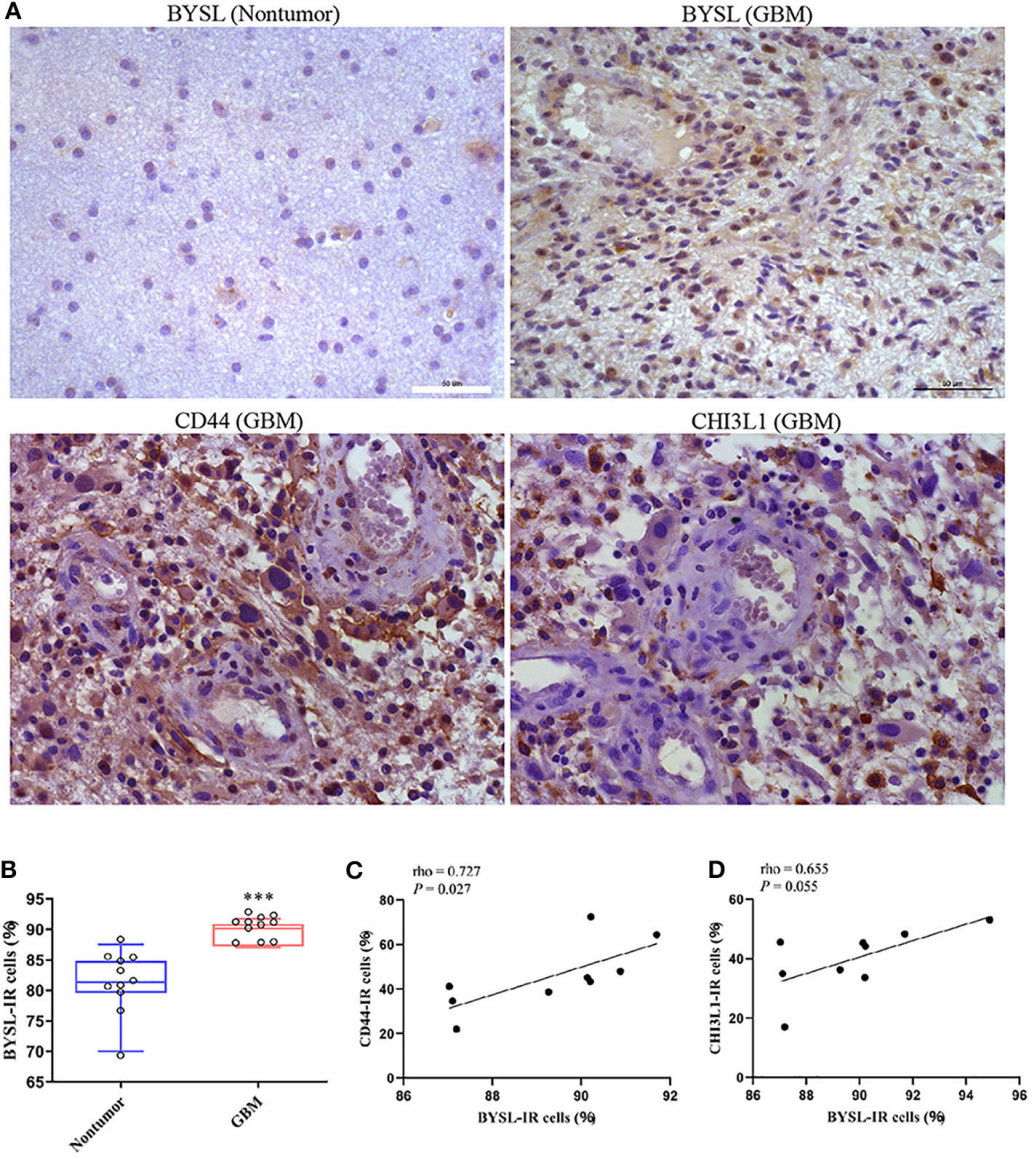
BYSL is upregulated in GBM and associated with the GBM mesenchymal subtype. Immunohistochemical analysis to measure the IR of BYSL, CD44, and CHI3L1 in non-tumor brain tissues and/or GBM tissues. **(A)** Representative images for the BYSL-, CD44-, and CHI3L1-IR staining. **(B)** Cell counts showing that the percentage of BYSL-IR cells was significantly increased in GBM tissues compared with non-tumor brain tissues. **(C,D)** Spearman correlation analysis showing an association of BYSL with markers of mesenchymal GBM subtype (CD44 and CHI3L1). Scale bars: 50 μm. ***P < 0.001.

The IR of BYSL was analyzed by immunohistochemistry followed by cell counting in non-tumor brain tissues and GBM tissues (n = 11 for each group). BYSL-IR was located in both cytoplasm and nucleus, and the percentage of BYSL-IR cells was significantly increased in GBM tissues (*P* < 0.001, Figures 9A,B). In addition, immunohistochemical data for CD44 and CHI3L1 were available for the GBM tissues (Figure 9A). Nine of the 11 GBM tissue samples were of mesenchymal subtype, as indicated by strong expression of CD44 and CHI3L1. BYSL was positively correlated with CD44 (rho = 0.727, *P* = 0.027) and CHI3L1 (rho = 0.655, *P* = 0.055) in the mesenchymal GBM subtype (Figures 9C,D). These findings suggest that BYSL is highly expressed in GBM, especially in the mesenchymal subtype.

## Discussion

In this study, we demonstrated that overexpression of BYSL promoted GBM cell migration/invasion and enhanced EMT. Silencing of BYSL showed the opposite effects. The GSK-3β/β-catenin signaling pathway was regulated by BYSL and was required for the promotion by BYSL of GBM cell migration/invasion and EMT. In addition, high expression of BYSL was found in GBM tissues and was positively correlated with mesenchymal markers CD44 and CHI3L1. Collectively, these results suggest that BYSL promotes GBM cell migration, invasion, and EMT through the GSK-3β/β-catenin pathway.

BYSL is upregulated in reactive astrocytes in response to brain injury or inflammation (32) and promotes liver cancer cell survival and tumorigenesis (10). In addition, BYSL promotes the growth and invasion of prostate cancer cells (12). In agreement with these findings, the current study showed that BYSL promoted the migration and invasion of GBM cells. Thus, BYSL is generally involved in the malignant progression of cancers.

The acquisition of EMT causes cell morphology to switch from a non-polar epithelial phenotype to a mesenchymal phenotype that is conducive to migration. This transition plays an important part in the infiltration and metastasis of tumor cells (13, 33). A number of epithelial and mesenchymal biomarkers are used to assess EMT in GBM cells (13). Our study demonstrated that knockdown of BYSL suppressed the expression of mesenchymal markers β-catenin and N-cadherin, and enhanced the expression of epithelial marker E-cadherin in GBM cells. Overexpression of BYSL showed the opposite effects. In addition, the role of BYSL in promoting EMT was further confirmed in a patient-derived GSC cell line. These results suggest that BYSL promotes EMT in GBM cells.

β-catenin is not only a hallmark of EMT but also an effector of the WNT/β-catenin signaling pathway (28, 34, 35). It has been suggested that WNT signaling contributes to mesenchymal transition, migration, and invasion in glioma cells (17, 19). GSK-3β is an important component of the axin degradation complex that determines β-catenin subcellular localization and activity (31, 35). Here, we found that overexpression of BYSL led to a significant increase in the phosphorylation of GSK-3β and the nuclear distribution of β-catenin. In line with this, the activity of β-catenin and the transcription of its target genes were significantly decreased in GBM cells when BYSL was downregulated. A selective GSK-3β inhibitor (36), 1-Az, could partially reverse these effects. These findings indicate that GSK-3β activity is required for BYSL-mediated β-catenin signal transduction in GBM cells.

GSK-3β is an AKT substrate, and AKT/GSK-3β signaling is known to be involved in EMT (37, 38). We found that inhibiting GSK-3β using 1-Az partially reversed the decrease in cell migration/invasion and EMT caused by BYSL downregulation, indicating that GSK-3β activity is required for the promotion by BYSL of migration, invasion, and EMT in GBM cells. As AKT could affect EMT directly or through GSK-3β (37), further investigations are needed to elucidate the role of BYSL in regulating AKT activity in GBM cells.

BYSL is highly expressed in liver cancer, in ovarian cancer tissues, and in prostate cancer cells near the peripheral nerves. Here, we found an upregulation of BYSL-IR in GBM. These results suggest that high expression of BYSL may be universally found in different cancer types. More importantly, BYSL showed positive correlations with CD44 and CHI3L1 in GBM. Both of these molecules are markers of the mesenchymal subtype of GBM (15, 39), which is characterized by a higher percentage of necrotic cells and associated inflammation (15). Thus, these results provide further evidence for the association of BYSL with the highly invasive features of the mesenchymal GBM subtype.

In this study, we used a previously validated siRNA to knock down BYSL (10). Although downregulation of BYSL had significant effects on cell migration, invasion, and EMT, the differences were small or the variation was large for some data. This may have been caused by the limitations of RNA interference. We also attempted experiments in a stable cell line mediated by shRNA lentivirus, but the cells stably silencing BYSL grew slowly or died, so they could not be used for functional experiments. An inducible shRNA system should be established for BYSL loss-of-function experiments in the future.

In summary, our results demonstrate that high levels of BYSL in GBM promote cell migration, invasion, and EMT *via* the GSK-3β/β-catenin signaling pathway. These data suggest that BYSL could serve as a biomarker for the invasive subtype of GBM and as a target for the development of anti-GBM drugs.

## Data Availability Statement

All datasets generated for this study are included in the article/Supplementary Material.

## Ethics Statement

The studies involving human participants were reviewed and approved by the Ethics Committee of Xuzhou Medical University. The patients/participants provided their written informed consent to participate in this study.

## Author Contributions

SG, ZS, and RY conceived the study, participated in its design, and drafted the manuscript. ZS, JZ, and YHW performed the *in vitro* experiments. CL established the GSC cell line. TZ and QM did the experiments related to clinical samples. MP, FY, and YW participated in data analysis. All the authors read and approved the final manuscript.

## Conflict of Interest

The authors declare that the research was conducted in the absence of any commercial or financial relationships that could be construed as a potential conflict of interest.
