# Supplementary material for: BYSL Promotes Glioblastoma Cell Migration, Invasion, and Mesenchymal Transition Through the GSK-3β/β-Catenin Signaling Pathway
Source: Front Oncol. 2020 Oct 15;10:565225. doi: 10.3389/fonc.2020.565225 (PMC7593785; doi:10.3389/fonc.2020.565225)
Supplement: Supplementary file 1 [file Data_Sheet_1.DOCX]

**Supplementary figures and legends**

**
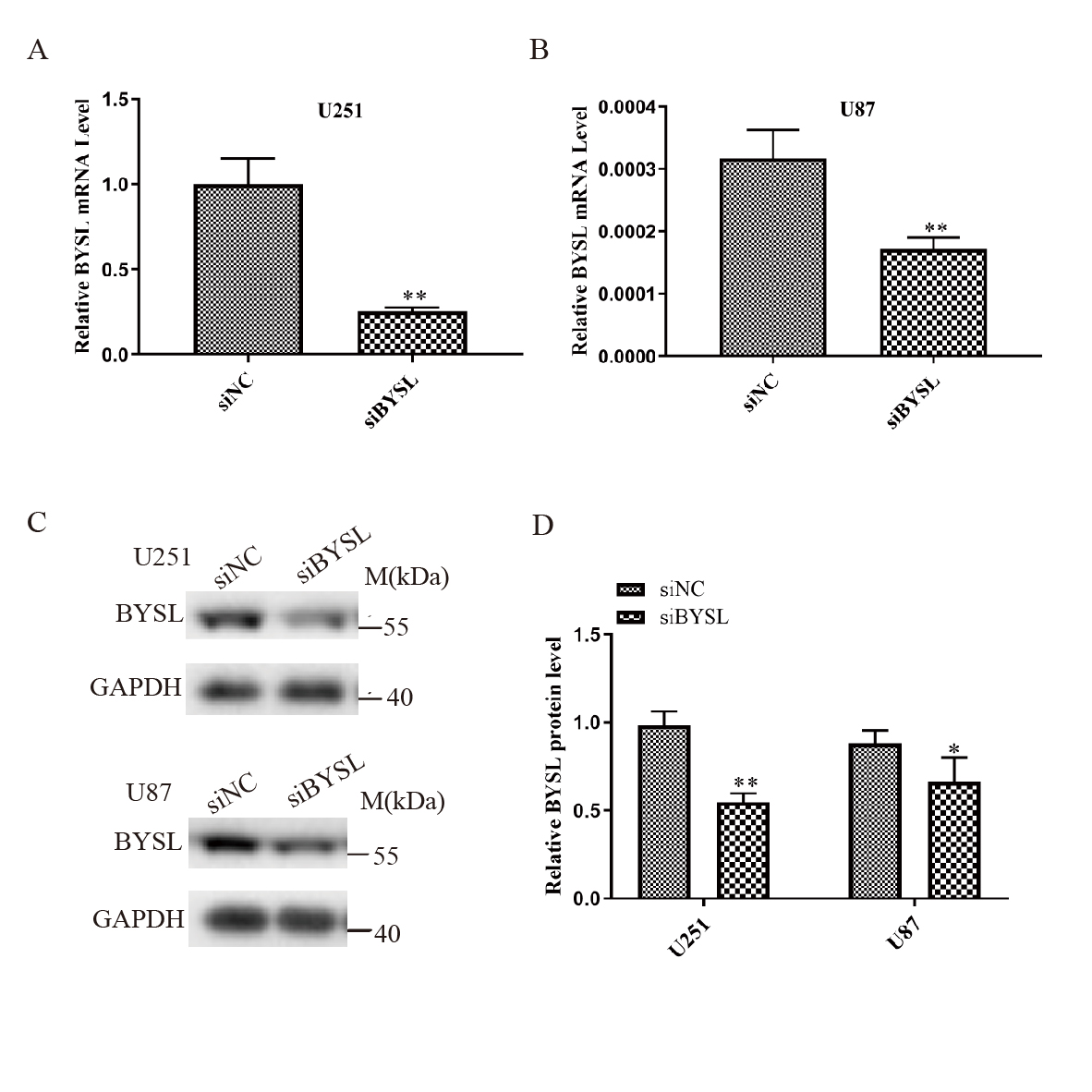
**

**Figure S1. The efficiency of BYSL downregulation in glioma cells** (**A**-**B**) The qRT-PCR assay showed that BYSL siRNA significantly downregulated the BYSL mRNA levels in both U251 and U87 cells. (**C**-**D**) Western blot analysis confirmed the downregulation efficiency of BYSL at protein levels in U251 and U87 cells. Representative blot images are shown in **C**, and quantification graph is shown in **D**. **P* < 0.05, ***P* < 0.01.


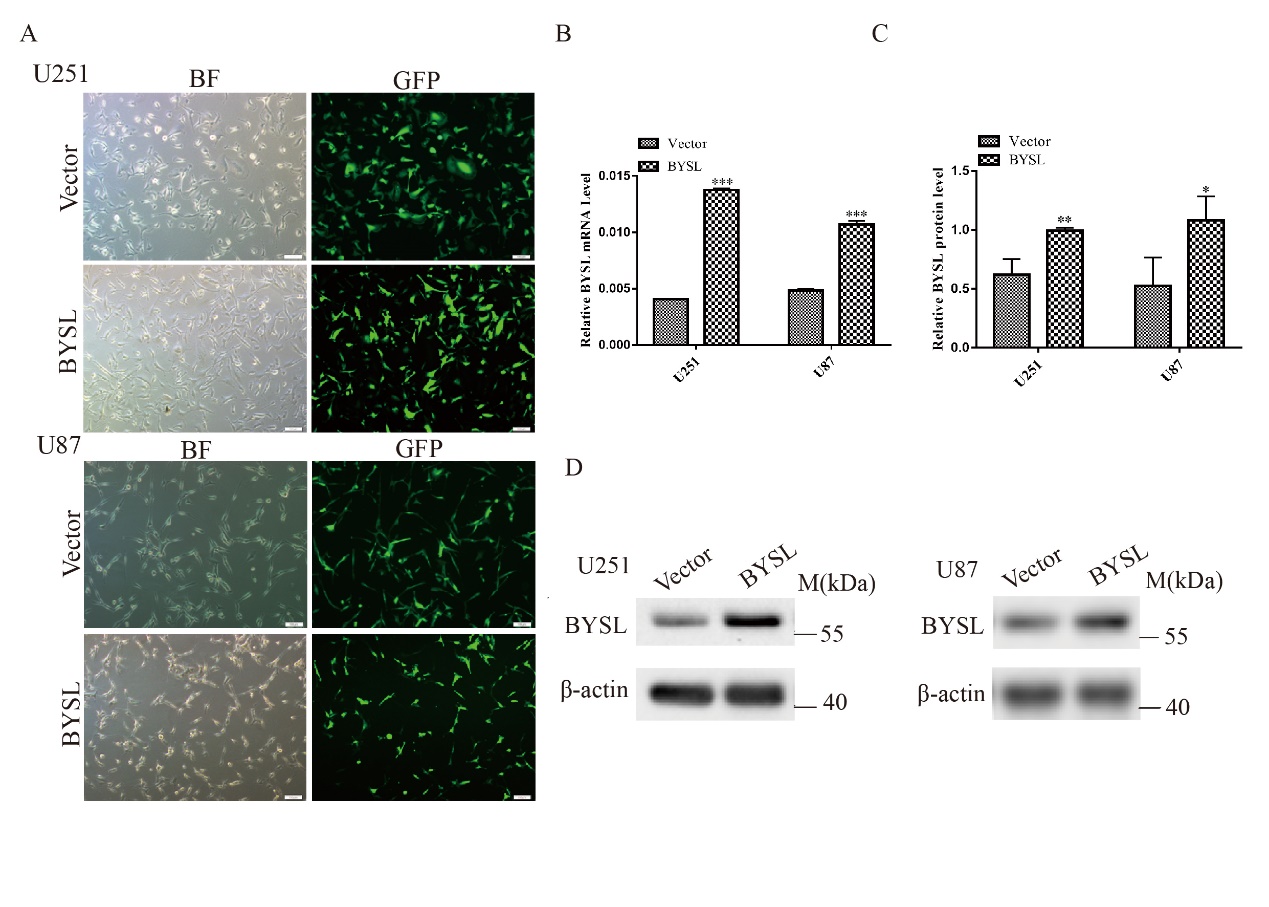


**Figure S2. The efficiency of BYSL overexpression in glioma cells** (**A**) Representative bright field (BF) and fluorescent field (GFP) images of Vector and BYSL-overexpressing U251and U87 cells. Scale bar: 100 μm. (**B**-**D**) The qRT-PCR (**B**) and Western blot (**C-D**) analyses showed that BYSL was successfully overexpressed in U251 and U87 cells. **P* < 0.05, ***P* < 0.01, ****P* < 0.001.

**
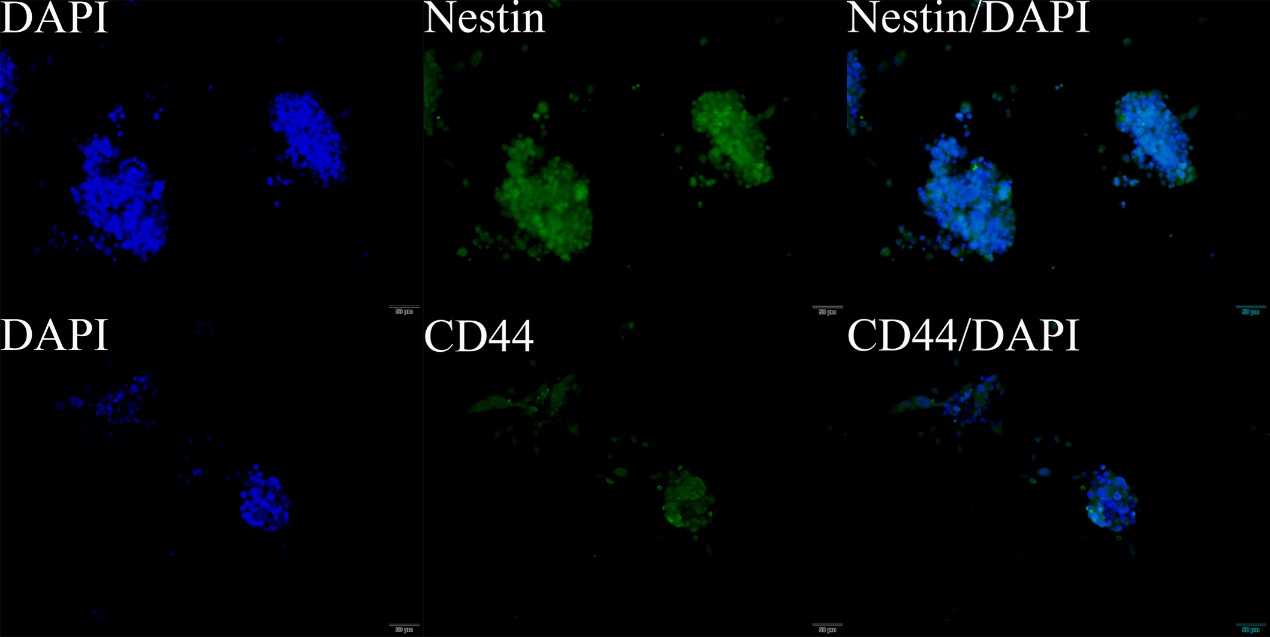
**

**Figure S3. Molecular characteristics of of GSC cells** Immunofluorescence staining was performed in a patient-derived GSC cell line (GSC-F). The GSC-F cells were observed to be positive for CD44 and Nestin, two stem cell markers. DAPI is a nuclear dye used as counterstain in immunofluorescence. Scale bar: 50 μm.
