# Supplementary material for: BYSL Promotes Glioblastoma Cell Migration, Invasion, and Mesenchymal Transition Through the GSK-3β/β-Catenin Signaling Pathway
Source: Front Oncol. 2020 Oct 15;10:565225. doi: 10.3389/fonc.2020.565225 (PMC7593785; doi:10.3389/fonc.2020.565225)
Supplement: Supplementary file 2 [file Data_Sheet_2.docx]

**Table S1** Clinico-pathological information for the studied subjects

| Case No. | Code No. | Gender | Age (years) | Used for | WHO Grade |
| --- | --- | --- | --- | --- | --- |
| 1 | 940512 | F | 49 | IHC | Nontumor |
| 2 | 946454 | M | 63 | IHC | Nontumor |
| 3 | 961081 | M | 10 | IHC | Nontumor |
| 4 | 963021 | M | 48 | IHC | Nontumor |
| 5 | 964460 | F | 52 | IHC | Nontumor |
| 6 | 971400 | F | 30 | IHC | Nontumor |
| 7 | 972078 | F | 49 | IHC | Nontumor |
| 8 | 986865 | M | 38 | IHC | Nontumor |
| 9 | 1004728 | M | 63 | IHC | Nontumor |
| 10 | 1024827 | F | 46 | IHC | Nontumor |
| 11 | 1095392 | M | 32 | IHC | Nontumor |
| 12 | 944968 | M | 62 | IHC | Grade IV |
| 13 | 966838 | M | 50 | IHC | Grade IV |
| 14 | 1053091 | M | 45 | IHC | Grade IV |
| 15 | 1075388 | M | 58 | IHC | Grade IV |
| 16 | 1086721 | F | 64 | IHC | Grade IV |
| 17 | 1088070 | F | 58 | IHC | Grade IV |
| 18 | 1145682 | M | 64 | IHC | Grade IV |
| 19 | 1059421 | M | 60 | IHC | Grade IV |
| 20 | 1073896 | F | 62 | IHC | Grade IV |
| 21 | 1184604 | F | 34 | IHC | Grade IV |
| 22 | 1507855 | M | 46 | IHC | Grade IV |

**Note:** F, Female; M, Male; IHC, immunohistochemistry; WHO, World Health Organization

**Table S2** The forward and reverse primers of genes in this study

| Gene | Forward primer | Reverse primer |
| --- | --- | --- |
| BYSL | 5'-CTGGTTCAAAGGGATCCTGA-3' | 5'-AGTCGCAGGAAGATGCTGTT-3' |
| β-actin | 5'-CCAACCGCGAGAAGATGA-3' | 5'-CCAGAGGCGTACAGGGATAG-3' |
| β-catenin | 5'-CCAGCCGACACCAAGAAG-3' | 5'-CGAATCAATCCAACAGTAGCC-3' |
| E-cadherin | 5'-GCTGGACCGAGAGAGTTTCC-3' | 5'-CGACGTTAGCCTCGTTCTCA-3' |
| N-cadherin | 5'-CCTCAGTCAACTGCAACCGT-3' | 5'-TGGGTCCTGAGCAGTGAATG-3' |
| Slug | 5'-CAAGGACCACAGTGGCTCAG-3' | 5'-CGCAGTGCAGCTGCTTATGT-3' |
| Vimentin | 5'-AAATGGCTCGTCACCTTCGT-3' | 5'-CAGCTTCCTGTAGGTGGCAA-3' |
| Twist-1 | 5'-TCGGACAAGCTGAGCAAGAT-3' | 5'-TCCATCCTCCAGACCGAGAA-3' |
| Twist-2 | 5'-AAGATCCAGACGCTCAAG-3' | 5'-GGTCATCTTATTGTCCATCTC-3' |
| MMP7 | 5'-GCTCAGGACTATCTCAAG-3' | 5'-ACATTCCAGTTATAGGTAGG-3' |
| Survivin | 5'-ACCGCATCTCTACATTCAAG-3' | 5'-CAAGTCTGGCTCGTTCTC-3' |
